# Software for near-real-time voltammetric tracking of tonic neurotransmitter levels *in vivo*

**DOI:** 10.3389/fnins.2022.899436

**Published:** 2022-09-23

**Authors:** Abhinav Goyal, Sangmun Hwang, Aaron E. Rusheen, Charles D. Blaha, Kevin E. Bennet, Kendall H. Lee, Dong Pyo Jang, Yoonbae Oh, Hojin Shin

**Affiliations:** ^1^Mayo Clinic Medical Scientist Training Program, Mayo Clinic, Rochester, MN, United States; ^2^Department of Neurologic Surgery, Mayo Clinic, Rochester, MN, United States; ^3^Department of Biomedical Engineering, Hanyang University, Seoul, South Korea; ^4^Division of Engineering, Mayo Clinic, Rochester, MN, United States; ^5^Department of Physiology and Biomedical Engineering, Mayo Clinic, Rochester, MN, United States

**Keywords:** cyclic voltammetry, tonic neurotransmitters, electrochemistry software, computational neuroscience, signal processing

## Abstract

Tonic extracellular neurotransmitter concentrations are important modulators of central network homeostasis. Disruptions in these tonic levels are thought to play a role in neurologic and psychiatric disease. Therefore, ways to improve their quantification are actively being investigated. Previously published voltammetric software packages have implemented FSCV, which is not capable of measuring tonic concentrations of neurotransmitters *in vivo*. In this paper, custom software was developed for near-real-time tracking (scans every 10 s) of neurotransmitters’ tonic concentrations with high sensitivity and spatiotemporal resolution both *in vitro* and *in vivo* using cyclic voltammetry combined with dynamic background subtraction (M-CSWV and FSCAV). This software was designed with flexibility, speed, and user-friendliness in mind. This software enables near-real-time measurement by reducing data analysis time through an optimized modeling algorithm, and efficient memory handling makes long-term measurement possible. The software permits customization of the cyclic voltammetric waveform shape, enabling experiments to detect a specific analyte of interest. Finally, flexibility considerations allow the user to alter the fitting parameters, filtering characteristics, and size and shape of the analyte kernel, based on data obtained live during the experiment to obtain accurate measurements as experimental conditions change. Herein, the design and advantages of this near-real-time voltammetric software are described, and its use is demonstrated in *in vivo* experiments.

## Introduction

Neurotransmitters such as dopamine and serotonin are responsible for mediating a wide array of neurologic functions, from memory to motivation (Ungerstedt and Pycock, 1974; Budygin et al., 2000; Arbuthnott and Wickens, 2007; Moran et al., 2018). Neurotransmitter release from synaptic vesicles is divided into phasic release and tonic release. Natural phasic release *in vivo* occurs when neurons burst fire at high frequencies to encode salient stimuli through their postsynaptic effects (Atcherley et al., 2015). In contrast, tonic release of neurotransmitters refers to neurotransmitter release via pacemaker-like firing (Ungerstedt, 1991; Atcherley et al., 2015). This background firing serves to maintain extracellular neurotransmitter levels within a certain concentration range, also called the “basal” or “tonic” concentration. These tonic neurotransmitter levels serve a wide range of functions; for example, tonic firing of dopaminergic neurons in the substantia nigra maintains a basal concentration of dopamine in the dorsal striatum to modulate basal ganglia excitability (Beeler et al., 2010). Deviations in concentrations of these neurotransmitters from their normal, homeostatic ranges is thought to underlie many neurologic and psychiatric diseases, including Parkinson’s disease, Alzheimer’s disease, schizophrenia, and depression (Nyholm, 2007; Guthrie et al., 2009; Grace, 2016). Therefore, it is of vital importance to develop and optimize techniques for measuring both phasic release and tonic levels of these neurotransmitters *in vivo.*

Previously published software packages to support specific types of voltammetric measurements of neurotransmitters *in vivo* include High Definition Cyclic Voltammetry (HDCV) (Bucher et al., 2013), Wireless Instantaneous Concentration Sensor software (WINCSware) (Lee et al., 2017), and Demon Cyclic Voltammetry (Demon CV) (Yorgason et al., 2011). These software packages are presently designed to be used with electrochemical instrumentation that perform fast-scan cyclic voltammetry (FSCV). This voltammetric technique is limited to recording only phasic changes in neurotransmitter concentrations *in vivo* (Baur et al., 1988; Michael et al., 1998). To measure phasic changes with FSCV, a background subtraction algorithm is necessary to remove non-Faradaic capacitive current and other sources of noise (Howell et al., 1986). However, inherent to the application of this algorithm includes the subtraction of tonic concentrations of the analyte of interest, rendering these software packages incapable of recording tonic concentrations. Voltammetric and non-voltammetric techniques designed to measure tonic information have been outlined in depth in a recent review (Rusheen et al., 2020).

The most common technique used to measure tonic concentrations of analytes is microdialysis, which involves sampling the extracellular fluid directly from the anatomic space of interest and quantifying the analyte concentrations via high-performance liquid chromatography (Benveniste, 1989; Ungerstedt, 1991). Thus, microdialysis has the advantage of being highly chemoselective, allowing for accurate discrimination between structurally similar analytes such as dopamine and norepinephrine. However, microdialysis also has several drawbacks which render it inefficient and unsuitable for future clinical application. First, time is required for collection of a sufficient volume of dialysate for analysis, making the temporal resolution of the technique too low (minutes per sample) to record rapid neurological processes such as synaptic transmission (Ungerstedt, 1991; Chefer et al., 2009). Second, microdialysis requires continuous perfusion of the dialysis probe with buffered solution and extraction of the dialysate from the brain for laboratory analysis, making this technique unrealistic for human implantation. Finally, the dialysis probe is quite large (typical diameter of 200–300 μm and length of 1–2 mm), which leads to tissue damage, induction of gliosis, and disruption of axon terminals along the entire length of the dialysis membrane (Di Chiara et al., 1993; Blaha et al., 1996). Both of these latter effects can skew extracellular concentration measurements, leading to the concern that microdialysis underestimates actual analyte concentrations (Bungay et al., 2003; Oh et al., 2018).

Techniques designed for use in humans to track tonic levels of neurotransmitters should have a temporal resolution on the order of seconds to capture the effects of salient stimuli and must be compatible with fully implantable electrodes that are small enough to not induce deleterious tissue damage or inflammation. Techniques that fit these criteria are emerging, with many utilizing voltammetry (Oh et al., 2018; Rusheen et al., 2020). Our lab has developed and utilized a novel electrochemical technique utilizing cyclic square wave voltammetry to provide accurate estimations of tonic extracellular concentrations of both dopamine (Oh et al., 2018) and serotonin (Shin et al., 2021) down to the sub-nanomolar range repetitively in 10 s intervals. These voltammetry-based techniques work with carbon-fiber microelectrodes (CFMs), which are composed of chemosensitive tips that are 7–10 μm in diameter and typically 50–100 μm in length. Prior work has demonstrated that CFMs can be implanted chronically with minimal tissue damage and inflammation (Peters et al., 2004). Thus, voltammetric techniques lack many of the drawbacks inherent with microdialysis and seem to be more suitable for future clinical applications. Indeed, recent work has demonstrated the utility of cyclic voltammetry for applications such as mechanistic modeling of the mammalian response to drugs of abuse (Yuen et al., 2021).

Here, a detailed description and *in vivo* demonstration of custom-written software is provided to control, monitor, and analyze the results of voltammetric data. It can generate and administer a wide variety of novel voltammetric waveforms to the CFM for identification of multiple neurochemical species. Compared to previous software platforms designed to implement FSCV, the software presented here permits measurements of tonic concentrations of neurotransmitters *in situ* both *in vitro* and *in vivo.* Because the analyte electrochemical current data is immediately analyzed and displayed, users can track how the tonic level of neurotransmitters behave and change in response to external stimuli in real-time.

## Materials and equipment

### General specifications

The developed software was based within a MATLAB Application Designer GUI linked to several MATLAB helper scripts for data processing. This software runs on a fixed rate timer with a 10 s execution period. The setup consisted of a desktop running the software interfaced to an NI-DAQ board (USB-6363, National Instruments, Austin, TX, USA) that was physically connected to the CFM through a transimpedance pre-amplifier (AD549, Analog Devices, Norwood, MA, USA), with an analog gain of 2,000 nA/V (500 kΩ) (Oh et al., 2018). Electrochemical current data from the CFM was sampled by the NI-DAQ board at 2 MHz and 16-bit depth and is referenced against an Ag/AgCl reference electrode placed within the beaker (*in vitro*) or contralateral cortex (*in vivo*). All data and figures were acquired and produced using a Windows desktop with an i5-9500, 3 GHz processor with 16 GB of RAM. Use of this software requires a MATLAB license with 3 toolboxes: the Parallel Processing toolbox for speeding up exponential background fitting, the Data Acquisition toolbox to enable communication with DAQ board hardware, and the Curve Fitting toolbox for exponential background fitting. The technology contained within this manuscript has been licensed to a company that is partly owned by WINCS International. The company is out from the Mayo Neural Engineering Laboratories. We have recently received a Small Business Innovation Research grant from NIH to commercialize this technique. Our intent is to commercialize this software together with electrochemical sensing hardware to allow researchers access to the technology contained within.

## Methods

### Monitoring tonic neurotransmitter concentrations

**FIGURE 1:**
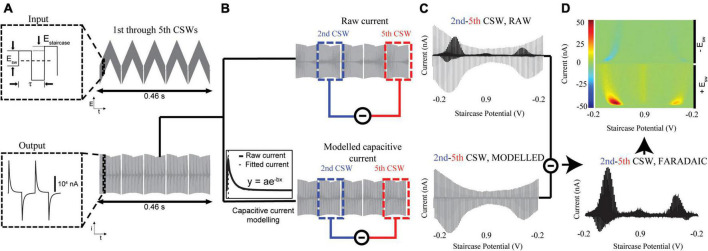
Extraction of tonic neurotransmitter concentrations. **(A)** *Top:* The input cyclic square waveform, consisting of square waveforms superimposed on staircase waveforms (5 shown), is applied to the CFM. This rapidly oxidizes and reduces electroactive species around the CFM, generating current. *Bottom:* The output current is digitized and sent to MATLAB for processing. The remaining steps **(B–D)** are performed by the software. **(B)** The total current (*Top*) consists of the non-Faradaic capacitive current and the Faradaic current of interest. The modeled capacitive current (*Bottom)* can be fit with MATLAB and subtracted away to yield the Faradaic current generated at each square wave. **(C)** *Top:* The 5th CSW is subtracted from the 2nd to remove the majority of the non-Faradaic current. *Bottom:* The rest of the non-Faradaic current is modeled by fitting the capacitive currents of the 2nd and 5th CSWs. The final vestiges of non-Faradaic current are eliminated by subtracting this modeled capacitive current from the true 2nd-5th CSW signal. **(D)** (*Bottom)* What remains is the tonic Faradaic signal present at the 2nd CSW. This can then be projected onto a 3-D pseudocolor plot (*Top*) for visualization. Red represents oxidation currents and blue represents reduction currents. The left half of the color plot are currents from the upward square wave pulses, and the right half are currents from the downward pulses.

Multi-Cyclic square wave voltammetry is a powerful new voltammetric technique that can measure tonic concentrations of analytes such as the neurotransmitters dopamine and serotonin *in vivo* with high sensitivity and selectivity (Oh et al., 2018). Our software employs multiple cyclic square wave voltammetry theory, which combines two electrochemical methods, cyclic square wave voltammetry and multi-scan voltammetry (Figure 1). Cyclic square wave voltammetry superimposes a series of square waves onto a staircase pattern (Figure 1A). Analyte molecules are oxidized and reduced by a square wave at each staircase voltage, leading to a series of redox reactions that improves sensitivity beyond waveforms that oxidize and reduce with a single triangular waveform sweep (e.g., FSCV). The current released by these redox reactions is measured by the recording electrode and digitized by a DAQ board to be read and displayed in our software. Figure 1A displays the input waveform (top) and the corresponding output current data (bottom). The nomenclature used in this report was adopted from Helfrick and Bottomley (2009). The waveform parameters, Esw, Estaircase, τ, gap, and repetition frequency, are 0.4 V, 0.025 V, 1 ms, 2 ms, and 0.1 Hz, respectively.

A side-effect of applying square wave voltages is the formation of an electric double layer at the surface of the CFM, which causes the system to behave as a capacitor. Because total stored charge is equal to capacitance multiplied by voltage, the capacitive current is equal to $I_{c}=\frac{d}{d\,t}\,\left(C\,V\right)=C\,\frac{d\,V}{d\,t}$. It can therefore be seen that the sharp increase in voltage seen in square waveforms will generate a large capacitive current which can approach 20,000 nA in magnitude (Figure 1B, bottom). This capacitive current is much larger than the underlying Faradaic current arising from analyte redox events, and thus must be removed to extract tonic-level information. Capacitive currents are exponentials in both the charging and discharging phases, with different time constants per phase. Because the charging phase closely follows the voltage step caused by each square waveform, it appears well before electrochemical species have been oxidized. Thus, the Faradaic signal of interest appears only within the discharging phase, serving to slow the decay rate of the current (Figure 1B, bottom inset). The discharging phase of non-Faradaic capacitive currents can be modeled as $I=I_{0}\,e^{-\frac{t}{\tau }}$. The software dynamically fits the capacitive discharges of the CSWs with best-fit parameters chosen by minimizing root mean square error (RMSE). Subtracting the fitted capacitive signal from the raw signal will yield the underlying Faradaic current (Figures 1C,D).

The multi-scan voltammetry method takes advantage of the idea that successively applying CSWs to the electrode without allowing time for analytes to adsorb will gradually reduce the analyte present to be oxidized. After a certain number of CSWs, the analyte oxidation current will approach 0, and the current present within the scan will consist of only the non-Faradaic (capacitive) signal. Thus, the tonic Faradaic analyte current can be determined by subtracting off the final scan (non-Faradaic only) from an earlier scan (non-Faradaic + Faradaic). In the case of dopamine, it was determined that, by the 5th CSW, analyte current had approached 0. Thus, much of the non-Faradaic signal is subtracted off by subtracting the 5th CSW from the 2nd (Figure 1C, top).

However, due to drift at the electrode surface (Rodeberg et al., 2017), some non-Faradaic signal remains in the 2nd–5th CSW. Because of the non-constant nature of background drift, this non-Faradaic signal is not directly quantifiable. To remove these residual signals, the modeled capacitive discharges of the 5th CSW are subtracted from those of the 2nd to yield the remaining non-Faradaic component of the 2nd–5th CSW signal (Figure 1C, bottom). This simulated non-Faradaic signal is then subtracted from the true 2nd–5th CSW signal, which contains both Faradaic and non-Faradaic components, to isolate the tonic Faradaic signal present at the time of the 2nd CSW (Figure 1D, bottom). This Faradaic voltammogram can then be projected onto a 3-D pseudocolor plot for visualization of the oxidation (bottom, red), and reduction (top, blue) currents (Figure 1D, top).

### Waveform creation

**FIGURE 2:**
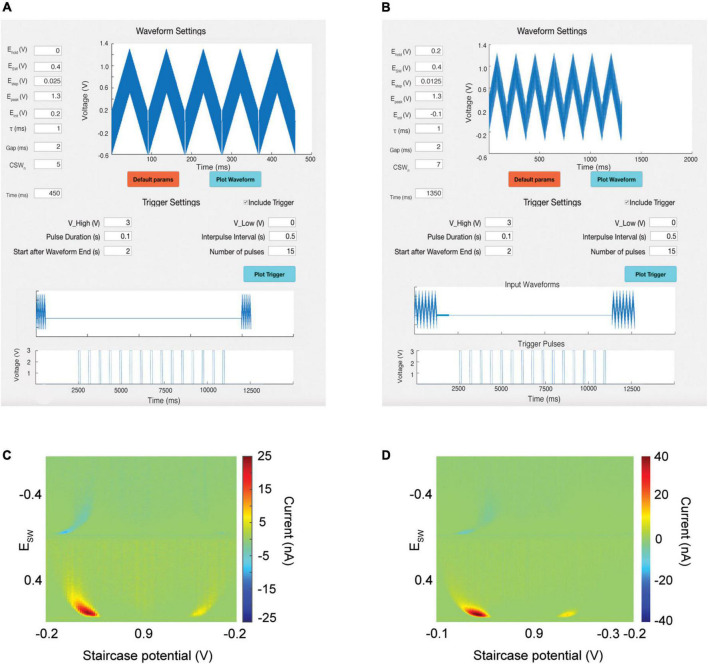
Waveform and trigger designer tool. Top: Users can create their own waveforms or choose from among a default set. Middle: Users can create their own trigger pulses to power external devices. The software time syncs the trigger pulses with input waveforms to avoid overlap. **(A)** M-CSWV waveform for detection of dopamine. **(B)** N-MCSWV waveform for detection of serotonin. **(C)** Output pseudocolor plot demonstrating detection of 500 nM of dopamine. **(D)** Output pseudocolor plot demonstrating detection of 100 nM of serotonin.

To allow flexibility for the user to apply a wide variety of waveforms for the detection of many different analytes, a waveform creation tool (Figure 2) was added to the software. The user will be able to set their own waveform parameters to generate waveforms of any shape:

**E**_Holding_ is the holding potential between successive CSWs.

**E**_sw_ is the height of the anodic and cathodic phases of the square wave.

**E**_Staircase_ controls how much each successive square wave increases in voltage (the slope of the staircase waveform).

**E**_Peak_ controls the maximum voltage achieved by the CSWs.

**E**_Initial_ is the initial voltage of the first square wave of each CSW.

τ controls the duration of one square wave for both anodic and cathodic phases.

**Gap** determines how long the holding potential is administered between successive CSWs.

**CSW**_n_ controls the number of CSWs per scan.

These parameters are all adjustable within the waveform designer tool (Figure 2, top). After the waveform is plotted, the Time box will populate with the total duration of the input waveform. The user can also choose from among a set of default parameters that have been previously validated by our team for specific analytes. Figure 2 demonstrates the use of this waveform creation tool to create the M-CSWV waveform (Figure 2A) and the N-MCSWV waveform (Figure 2B) for detection of tonic concentrations of dopamine and serotonin, respectively (Figures 2C,D).

### Gating and triggering

To allow the user to trigger external devices, such as electrical stimulators, without inducing artifact during neurochemical sensing, the software allows for the creation of trigger pulses (Figures 2A,B, middle). The user can adjust the amplitude, duration, start time, interpulse interval, and number of stimulation pulses. The software will then plot the proposed trigger sequence against the input waveform time schedule. The user can then ensure that the trigger sequence is designed to avoid overlap between the triggered stimulations and the waveforms. The waveform and the trigger sequence can be transmitted via different analog-out channels from the DAQ board. This ability has several important applications. First, this allows the software to interleave voltammetric scanning with electrical stimulation to ensure that stimulation artifacts do not interfere with the electrochemical signal. Second, this allows for the generation of non-standard pulse sequences that cannot be achieved with older analog stimulators, such as theta burst stimulation. Thus, the user will not need to set up a separate gating trigger apparatus to control external devices.

### Software execution

**FIGURE 3:**
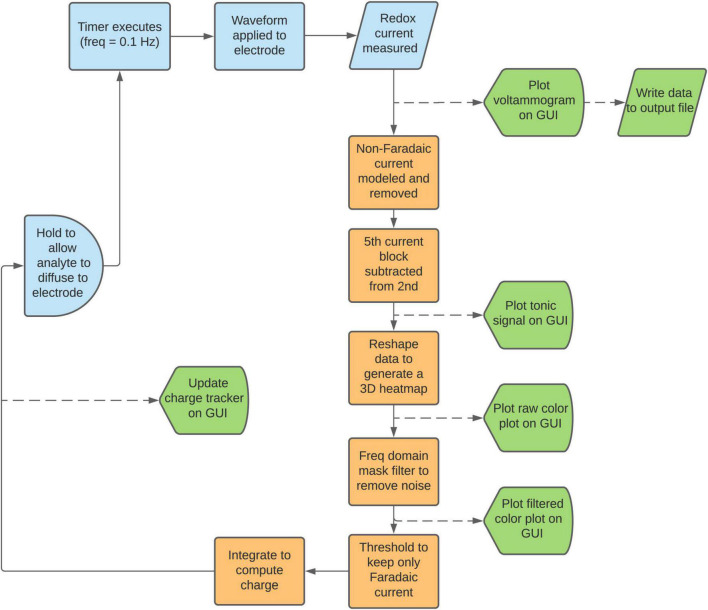
Data analysis flowchart. Starting from the upper left, the workflow proceeds as described in the manuscript. Squares denote processes, semicircles denote delay blocks, rhombi denote data I/O blocks, and pentagons denote display blocks. Processing steps that occur without user input are denoted in orange, and data that is plotted to the GUI (Figure 4) are denoted in green.

A flowchart of the software execution steps is outlined in Figure 3. Prior to beginning the experiment, the user chooses the input waveform. The user will be able to load a waveform from a default set or generate their own using the waveform designer tool (Figure 2). The timer program is by default set to execute every 10 s, but this is adjustable. Upon execution of the timer program, the input waveform is applied through the CFM, rapidly oxidizing and reducing the analytes present. The resulting current data from these redox reactions is passed from the CFM through a pre-amplifier into a DAQ board, which samples it at 2 MHz and transmits the digitized signal to MATLAB for processing.

To minimize the RAM used by the software, all raw data are converted from 32-bit doubles to 16-bit signed integers and are written to a standard text file at each scan. The fitting parameters and processed data are discarded after each scan, such that toggling of any parameters during the live data acquisition will not affect the data to be used for post-processing. This allows for considerable flexibility to try different parameters during post-processing. However, there is also an option for the user to save these parameters to a csv log file, which can be used in post-processing to replicate conditions during the live experiment, if desired. These data optimization steps allow for chronic experiments that can run for many hours without the risk of saturating computer memory. This is essential, as even just the raw data is very large. A 5-h experiment produces an output text file of 2.7 GB. Retaining these arrays within MATLAB’s active memory would slow down the CPU and increase the risk of crashing during long experiments.

### *In vivo* experiment

The *in vivo* proof-of-concept experiment was performed with a Sprague-Dawley male rat (250 g). The rat was taken from a batch of rats that was kept in social housing in an AAALAC accredited vivarium following a standard 12-h light/dark cycle at constant temperature (21°C) and humidity (45%) with *ad libitum* food and water. The present studies were approved by the Institutional Animal Care and Use Committee (IACUC), Mayo Clinic, Rochester. The NIH Guide for the Care and Use of Laboratory Animals guidelines (Department of Health and Human Services, NIH publication No. 86-23, revised 1985) were followed for all aspects of animal care.

The rat was anesthetized with urethane (1.5 g/kg, i.p., Sigma-Aldrich, St. Louis, MO, USA). After depth of anesthesia was confirmed with loss of hind limb nociceptive withdrawal response, it was fixed to a stereotactic surgical frame (David Kopf Instruments, Tujunga, CA, USA). A burr hole was drilled over the right dorsal striatum (stereotactic coordinates from bregma (Paxinos and Watson, 2006): AP: +1.2, ML: +2.0, DV: –4.5) for placement of the CFM. Another burr hole was drilled on the contralateral side for placement of the chlorinated Ag/AgCl reference electrode. The CFM was lowered into the dorsal striatum, and the M-CSWV signal was allowed to stabilize for 60 min. The DA selective terminal reuptake blocker nomifensine (20 mg/kg, i.p., Sigma-Aldrich, St. Louis, MO, USA) was injected and the tonic neurotransmitter signal was tracked for 1 h.

### Carbon-fiber microelectrode and reference electrode fabrication

CFMs were fabricated as described previously (Chang et al., 2013). Briefly, a carbon fiber strand was threaded through silica tubing and bonded with epoxy glue. The silica-CF construct was then bonded to a nitinol wire with silver paste. Polyamide tubing was used to ground most of the electrode, leaving only a bit of the silica exposed on one end, and a bit of the nitinol wire exposed on the other end. The reference electrode was prepared using 2 coated Ag wires. The ends of both wires were stripped to reveal the Ag interior. One of the stripped ends of both wires were connected to separate terminals of a 9V battery, and the other ends were placed in saline solution. This forms an electrochemical cell, which generates the half reaction Ag + Cl – > AgCl at the wire connected to the positive terminal of the battery. This treated end is then placed within the system to be recorded.

### Calibration of carbon-fiber microelectrodes

The output of the M-CSWV processing algorithm gives the analyte charge at each scan. In order to convert this charge into analyte concentration, the CFM must be calibrated using *in vitro* solutions of known analyte concentrations. For the *in vivo* experiment presented in this work, the CFM was calibrated using solutions of dopamine dissolved in Tris buffer. To construct the calibration curve, 100, 300, and 500 nM solutions were used, and the charge at each concentration was computed to generate a concentration vs. charge curve for the specific CFM. The charges at each scan from the *in vivo* experiment can then be fitted to this curve to estimate the concentration of analyte at the scan.

### Processing steps and user interface

**FIGURE 4:**
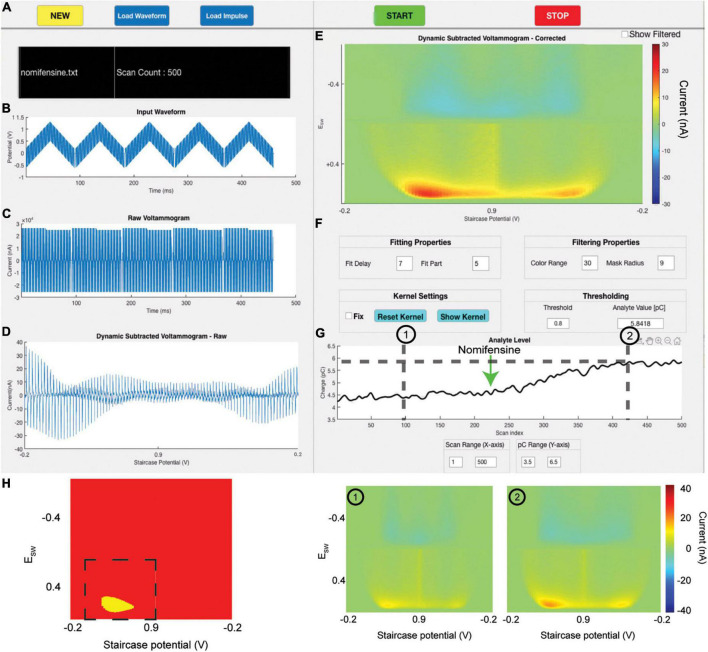
Software user interface. **(A)** Buttons to control the GUI, output text file name, and current scan count. **(B)** Plot of waveform applied to the electrode at the beginning of each scan. **(C)** The raw redox current trace response to the input waveform. **(D)** Non-Faradaic-subtracted voltammogram as a function of input voltage. **(E)** A 3D color plot of the non-Faradaic-subtracted voltammogram plotting input voltage on the *X*-axis, the amount that the input voltage is swept through with each square wave on the *Y*-axis, and current as the intensity. The bottom half plots oxidation currents, and the left half plots current response to the upward sweep of each square wave. **(F)** User-defined fitting, filtering, kernel, and thresholding parameters. **(G)** Charge trace which tracks the analyte charge computed for each scan. For this experiment, nomifensine was administered at scan 225. **(H)** Left: Real-time dopamine kernel used for charge calculation. Dashed line indicates the area used for charge computation. Right: 3D color plots of pre-nomifensine (scan 175) and post-nomifensine (scan 450) states.

Figure 4 displays the software user interface from a representative rat experiment. Panel 4A displays the buttons to operate the software, name of the output text file, and the current scan count.

The input voltammetric waveform is plotted in Figure 4B. The output raw current data can be visualized as a voltammogram by plotting output current vs. time (Figure 4C). The five blocks of the output voltammogram correspond to the five CSWs of the waveform. Within each block contains the current trace that resulted from the corresponding square wave. These current traces are theoretically specific to the analyte being measured. They consist of two parts: non-Faradaic (or “background”) current and Faradaic (or “analyte”) oxidation or reduction current. Sources of non-Faradaic current are diverse and discussed in depth elsewhere (Johnson et al., 2017, 2018).

The exponentially declining non-Faradaic current trace is fit with the “fit” function from MATLAB’s Curve Fitting toolbox and subtracted from the raw data, leaving behind only the Faradaic current. As explained previously, tonic levels are tracked by subtracting the current data present within the 5th Faradaic block from the current data in the 2nd one. This subtracted data therefore theoretically represents only the Faradaic current generated by the redox reactions at each square wave within the 2nd staircase, as previous work has demonstrated that this subtraction process also removes Faradaic current generated at the electrode surface (Oh et al., 2018). However, due to drift in the background current and other deviations in response over time, this process does not completely remove the non-Faradaic current component, but it does attenuate it to be below the Faradic component (Figure 4D). The resultant current data is projected onto a pseudo-color plot (Figure 4E).

Finally, to determine the total charge present at the electrode surface, the intensity of the color plot (i.e., the current) is integrated over both voltage and time. A thresholding algorithm is applied that dynamically zeros out contributions below 1 standard deviation (adjustable) above the average current response over all scans, creating a kernel (Figure 4H, left). By examining this kernel in real time, the user can adjust the threshold accordingly to ensure that the signal is being maximized while excluding as much noise as possible. The kernel is then averaged to produce a mean Faradaic charge value from oxidation or reduction of the analyte during that scan. This averaged charge is tracked continuously and plotted (Figure 4G), allowing for near real-time evaluation of the effects of behavioral responses or to exogenous stimuli such as electrical stimulation, drug administration, or tactile stimulation on analyte levels. Using the scan repetition rate, the *x*-axis of the plotted charge can be converted to time.

## Results

### User control and flexibility

**FIGURE 5:**
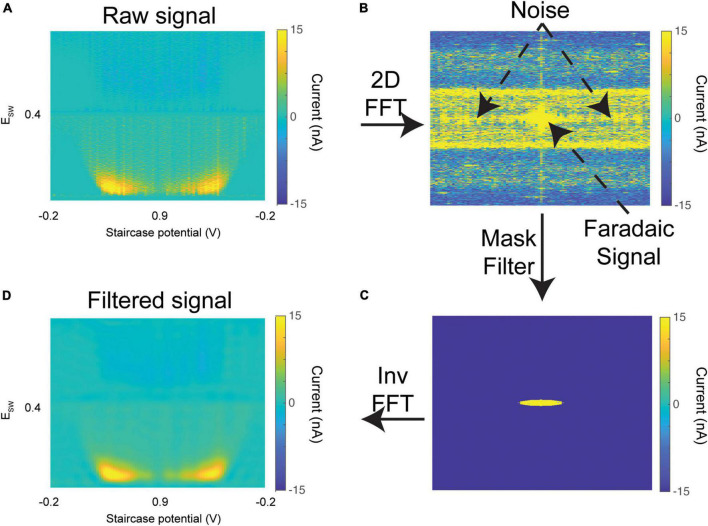
Mask filter. **(A)** Raw 3-D color plot of dopamine oxidation currents (similar to Figure 4E) from an *in vivo* experiment. **(B)** 2-D Fast Fourier transform of the raw color plot, with low frequencies shifted to the center. **(C)** An elliptical mask filter is applied which zeros out all contributions outside its boundary. **(D)** Inverse 2-D Fast Fourier transform to recover the color plot. The oxidation currents are preserved, while noise was effectively filtered out.

To provide the user a great degree of control over the live tracking process, the software provides several options to allow the user to dynamically alter background fitting, plotting, and mask filter parameters (Figure 4F). It should be noted that adjusting these parameters does not alter the data that is written to the output text file; thus, the raw data can be re-analyzed using any combination of parameters. This allows the user a great degree of flexibility to adjust parameters during live tracking. The fitting properties adjust how the background subtraction algorithm is performed. The filtering properties include setting the radius of the mask filter (Figure 5C) and changing the color bar range of the 3-D color plots (Figure 4E). Toggling on the “Fix” checkbox will hold the minimum threshold at its present level, rather than dynamically calculating a new threshold at each scan. If the “Fix” box is toggled on, pressing the “Reset Kernel” button will calculate a new threshold based on the previous 20 scans, and use that as the new fixed minimum threshold. Finally, the Threshold value controls the minimum current threshold for the dynamic thresholding algorithm. It sets the number of standard deviations above the mean current, below which all current contributions are zeroed out.

### Software optimization

The software described in this work has undergone many iterations and improvements since our first publication on tonic recordings (Oh et al., 2018). The MATLAB software has been optimized to maximize data processing speed, as the previous current data must be fully processed and plotted prior to the next timer program execution (i.e., each instance of current data must be processed in < 10 s). This was achieved by parallelizing the exponential fitting of each current trace using MATLAB’s Parallel Processing toolbox. The fitting process is essential in the measurement of the tonic concentrations of neurotransmitters (Oh et al., 2018). For real-time measurements, the fitting process was optimized to reduce the analysis time to allow users to run the software on a standard Windows desktop. Since the analysis time has been reduced, there is room for further increasing the temporal resolution or including additional real-time analyses depending on the experimental conditions in the future. In addition, the SNR of tonic neurotransmitter signals was improved by flexibly applying adaptive 2D FFT filters and thresholder kernels rather than temporal filters that are inappropriate for use in high-dimensional measurements. Finally, this iteration of the software includes many useful functions such as input waveform customization, a gating trigger to control external hardware, and flexibility for the user to modify fitting, filtering, and thresholding parameters.

### Filtering and charge computation

Sources of noise from the hardware or ambient environment often percolate through the signal. This can be seen in Figure 5A, where the color plot is contaminated with high-intensity broadband noise. A spatial frequency mask filter is optionally applied to the 2-D Fourier transform of the color plot (Figure 5B). The center of the FFT (low frequencies) corresponds to the signal, while the power present throughout the higher frequencies corresponds to noise. Since the color plot has non-equal length and width, an elliptical mask filter is applied (Figure 5C). The mask filter zeros out these higher frequency contributions while retaining the signal of interest. The filtered color plot is then extracted through an inverse 2-D Fourier transform. As can be seen in Figure 5D, the noise within the filtered color plot is significantly attenuated compared to the raw color plot.

To test whether this filtering process significantly affected the charge calculated within the signal, M-CSWV data from 8 random *in vivo* experiments (1 rat per experiment) conducted previously in our lab were analyzed. The mean analyte charge computed over the course of the experiment was compared between filtered and unfiltered data. We found that the filtering process does not significantly affect the charge calculated within the signal [*n* = 8 experiments; charge_*raw*_ (mean ± 95% CI) = 19.02 ± 2.1 pC; charge_filtered_ (mean ± 95% CI) = 18.43 ± 1.8 pC]. The radius of the mask is adjustable by the user to ensure that no component of the signal is zeroed out. In Figure 4E, the “show filtered” checkbox can be used to toggle between plotting the raw vs. mask-filtered color plots. Importantly, this filtered data is not saved to the output text file, so the raw data is not influenced by its use.

### *In vivo* experiment

In addition to outlining the technical specifications of our software, we also demonstrate its utility in an *in vivo* animal experiment. Figure 4 shows a single screenshot at scan 500 of an *in vivo* experiment assessing the effects of nomifensine administration on tonic dopamine levels in the rat dorsal striatum (see Supplementary Video 1 demonstrating software operation over all 500 scans). Nomifensine is a selective dopamine terminal reuptake inhibitor (Dubocovich and Zahniser, 1985), and its administration would be expected to slowly increase extracellular levels of dopamine over time. Nomifensine was administered at scan 225. The charge trace over the course of the experiment is shown in Figure 4G and is seen to track in near-real-time the expected increase in dopamine signal after nomifensine administration. The tonic dopamine increase after nomifensine administration is expected to peak at around 30 min after i.p. injection (Carboni et al., 1989), corresponding to scan 405. Pseudo color plots at scans 100 (pre-drug) and 425 (post-drug) are shown, as well as the kernel used to compute the charge (Figure 4H). An increase in the oxidation current (bottom left zone of activation) can be seen in the post-drug scan, corresponding to the increase in charge. A charge increase of approximately 1.6 pC, corresponding to a 100 nM (67%) increase in tonic extracellular levels of dopamine (determined via calibration with a 200 nM solution of dopamine *in vitro*), is seen, as one might expect after administration of nomifensine.

## Discussion

Here, we have outlined in detail our new software which enables tracking of tonic neurotransmitter levels in real time both *in vitro* and *in vivo*. This tonic tracking software was designed with speed, flexibility, and user-friendliness in mind. Systems-level optimizations, including parallelization of code execution where appropriate, enable the software to perform its entire processing pipeline in under 10 s, including sending the cyclic square waveform to the CFM, recording the output voltammogram, and transforming this raw voltammogram to an analyte concentration through filtering, capacitive current fitting, dynamic background subtraction, transformation to a pseudocolor plot, and post-calibration. This entire pipeline can be performed on a standard Windows desktop (this work was all done on an i5 processor with only 16 GB of RAM). In terms of flexibility, the software’s waveform creation tool allows users to customize pre-existing cyclic square waveforms (the software is preloaded with standard waveforms for detecting dopamine and serotonin) or generate their own for detection of specific analytes of interest. The software further supports gating and triggering by external hardware such as electrical stimulators, allowing for interleaved recording and stimulation to ensure that electrochemical recording is not contaminated by stimulation artifact. Finally, to maximize user-friendliness, the software was designed to work “out of the box,” assuming that MATLAB and the necessary toolboxes are available to the researcher. Barring any customization needs by the user, a dopamine or serotonin-specific waveform can be loaded in, and the software can be run with no further input by the user. However, in the interest of customizability and flexibility, the user is afforded extensive control over low-level parameters controlling capacitive fitting, filtering, and kernel determination. This software is therefore readily usable by and useful to all electrochemical researchers, from beginners to advanced.

Tonic extracellular neurotransmitter concentrations mediate a variety of physiologic phenomena, including network homeostasis and excitability. The current inability to track these concentrations on rapid time scales with high spatial resolution has prevented researchers from understanding the second-to-second effects of stimuli such as drug administration, electrical stimulation, or waking behavior on these tonic concentrations. This software supports application of cyclic square wave voltammetry, which enables highly sensitive detection of single analytes to the sub-nanomolar range with high temporal resolution (0.1 Hz scan rate) (Oh et al., 2018; Shin et al., 2021). This has enabled, for the first time, a more detailed and robust understanding of how these external stimuli affect tonic extracellular concentrations of analytes such as dopamine and serotonin on a rapid time scale. For example, recent work has employed cyclic square wave voltammetry to measure with high spatiotemporal resolution the effects of cocaine administration on tonic dopamine concentrations in the nucleus accumbens core (Yuen et al., 2021), and has used a modified N-shaped cyclic square waveform to enable highly specific recordings of tonic concentrations of serotonin (Shin et al., 2021). Overall, cyclic square wave voltammetry has the potential to provide an unprecedented level of insight into both physiologic and pathophysiologic neurotransmitter signaling. With the advent of novel cyclic voltammetry applications, the fact that our software is able to handle increasingly complex and diversified waveforms ensures its utility into the future.

When compared to previous techniques and software platforms that implement FSCV, the present software offers greater flexibility overall. In addition to the drawbacks of the *in vivo* microdialysis technique discussed previously (see Introduction), analysis of *in vivo* microdialysis cannot be performed in real-time, and must be performed *e situ* (i.e., samples must be taken to a laboratory, preventing neurotransmitter quantification in the moment). In contrast, our live tonic recording software allows the user to dynamically alter their experimental paradigms (such as electrical stimulation parameters) based on results from the current experiment. Other well-known software platforms, including HDCV, (Bucher et al., 2013) WINCSware, (Lee et al., 2017) and Demon CV (Yorgason et al., 2011), implement FSCV, which does not allow for application of cyclic square waveforms, and automatically performs rolling background subtraction. This, by definition, precludes measurement and analysis of tonic concentrations of neurotransmitters. The software described herein can apply a variety of cyclic square waveforms suitable for tonic measurements. Many previous publications that include cyclic square-wave voltammetry-based measurement of tonic neurotransmitter concentrations employed a software written in LabVIEW (Oh et al., 2018; Park et al., 2018; Shin et al., 2020). However, the reduced parallelization and optimization capabilities of the LabVIEW platform made real-time tracking impossible, as the cyclic square wave processing could not be performed in under 10 s. By switching to MATLAB and adding code execution optimizations, the current version of the software offers considerably enhanced speed, flexibility, and user-friendliness. Further, MATLAB is currently widely available to the majority of academic institutions, making our software simple to set up after the associated toolboxes have been downloaded.

Additionally, our *in vivo* proof-of-concept experiment demonstrates the software’s ability to track tonic neurotransmitter levels (with dopamine as an example) over time, as well as alterations in these levels in response to drug administration. While microdialysis can monitor gross alterations in tonic concentrations, its low temporal resolution makes characterizing the temporal dynamics of rapid responses to stimuli such as brief or intermittent electrical stimulation nearly impossible. In contrast, voltammetric analysis allows for direct visualization of these alterations in near real time, allowing researchers to confidently compare tonic responses between different drugs or stimulation conditions for the first time. This aspect of the software has a myriad of potential applications for exploring the real-time temporal dynamics of tonic neurotransmitter level alterations in response to various stimuli, such as drugs of abuse (Yuen et al., 2021), social/appetitive behaviors, and seizures, among many others.

In conclusion, this software employs techniques which, in combination with CFMs, boast higher spatiotemporal resolution, sensitivity, and user-friendliness than other techniques for tracking tonic neurotransmitter levels, such as microdialysis. Efficient systems-level optimizations that ensure processing time and storage use are kept to a minimum allow this software to be run for extended periods of time on a standard lab computer. Given the importance of tonic levels of neurotransmitters to both normal and abnormal physiology and behavior, in the future, we expect these voltammetric waveforms and this software to be actively applied to explore these tonic dynamics.

## Data availability statement

The raw data supporting the conclusions of this article will be made available by the authors, without undue reservation.

## Ethics statement

The animal study was reviewed and approved by the Mayo Clinic IACUC.

## Author contributions

AG and SH worked on the manuscript, code, and data analysis. KL and DJ developed the study conception and design. AR, CB, KB, and YO provided valuable guidance and manuscript edits. HS oversaw project execution and provided valuable guidance and manuscript edits. All authors reviewed the results and approved the final version of the manuscript.
